# A Novel Method for Measuring Anterior Segment Area of the Eye on Ultrasound Biomicroscopic Images Using Photoshop

**DOI:** 10.1371/journal.pone.0120843

**Published:** 2015-03-24

**Authors:** Zhonghao Wang, Xuanwei Liang, Ziqiang Wu, Jialiu Lin, Jingjing Huang

**Affiliations:** 1 State Key Laboratory of Ophthalmology, Zhongshan Ophthalmic Center, Sun Yat-sen University, Guangzhou, China; 2 Center for Advanced Eye Care, Carson City, Nevada, United States of America; Glasgow University, UNITED KINGDOM

## Abstract

**Purpose:**

To describe a novel method for quantitative measurement of area parameters in ocular anterior segment ultrasound biomicroscopy (UBM) images using Photoshop software and to assess its intraobserver and interobserver reproducibility.

**Methods:**

Twenty healthy volunteers with wide angles and twenty patients with narrow or closed angles were consecutively recruited. UBM images were obtained and analyzed using Photoshop software by two physicians with different-level training on two occasions. Borders of anterior segment structures including cornea, iris, lens, and zonules in the UBM image were semi-automatically defined by the Magnetic Lasso Tool in the Photoshop software according to the pixel contrast and modified by the observers. Anterior chamber area (ACA), posterior chamber area (PCA), iris cross-section area (ICA) and angle recess area (ARA) were drawn and measured. The intraobserver and interobserver reproducibilities of the anterior segment area parameters and scleral spur location were assessed by limits of agreement, coefficient of variation (CV), and intraclass correlation coefficient (ICC).

**Results:**

All of the parameters were successfully measured by Photoshop. The intraobserver and interobserver reproducibilities of ACA, PCA, and ICA were good, with no more than 5% CV and more than 0.95 ICC, while the CVs of ARA were within 20%. The intraobserver and interobserver reproducibilities for defining the spur location were more than 0.97 ICCs. Although the operating times for both observers were less than 3 minutes per image, there was significant difference in the measuring time between two observers with different levels of training (*p*<0.001).

**Conclusion:**

Measurements of ocular anterior segment areas on UBM images by Photoshop showed good intraobserver and interobserver reproducibilties. The methodology was easy to adopt and effective in measuring.

## Introduction

Measurements of the anterior segment are important in the assessment of a variety of ocular diseases, especially in the diagnosis and follow up of angle closure. Ultrasound biomicroscopy (UBM) can provide high-definition cross-sectional images of the anterior chamber, iris and angle structures, retroiridial structures, and ciliary processes, allowing reliable and repeatable quantitative measurements on ocular anterior segment [1,2].

The built-in software of the UBM device provides limited linear measurements which only give partial information about the anterior segment. Beyond static and linear measurements, changes in the configuration and volume of intraocular structures have been found to be physiological risk factors of various ocular diseases [3]. The iris and its relationship with the anterior chamber, posterior chamber, and the angle recess area, in particular, are recognized as important aspects into the pathogenesis of angle closure [4–6]. Some image analysis software have been developed recently [7,8]. However, due to the irregularity of the boundaries of the anterior segment, it is difficult to perform area measurements using images generated by UBM. Such study may provide important insight into the pathogenesis of anterior segment diseases.

Photoshop (Adobe Photoshop, Adobe Systems Inc, CA, USA) is a widely-used software which provides simple, effective, and time-saving methods for two-dimension image processing. In the current study, we describe a novel algorithm to quantify the anterior segment area in UBM images using Photoshop. The interobserver and intraobserver reproducibilities of the measurements are also assessed.

## Methods

### Participants

All research involving human participants were approved by the Zhongshan Ophthalmic Center, Sun Yat-sen University Institutional Review Board (IRB), and all clinical investigation were conducted according to the principles expressed in the Declaration of Helsinki. Written informed consent was obtained from the participants.

Images were collected with the Suoer panoramic UBM (Suoer Electonic Ltd, Model SW-3200L) in the Zhongshan Ophthalmic Center. Twenty healthy volunteers with wide angles and twenty patients with narrow or closed angles were consecutively recruited. The inclusion criteria of normal subjects were visual acuity >0.5; spherical correction <3.0 D and cylindrical power <2.0 D; no abnormal finding on slit lamp and fundus examinations; wide and open anterior chamber angle graded by gonioscopy; no history of eye injury, previous intraocular surgery, corneal opacity, or any type of glaucoma. The diagnoses of the twenty consecutive patients with narrow or closed angle were primary angle closure suspect, primary angle closure, and primary angle closure glaucoma, as defined using the ISGEO classification [9].

### Ultrasound biomicroscopy (UBM) images and analysis

UBM examinations were performed with the patients lying in a supine position in a dimly lit room (illumination 60–70 Lux, Model TES-1339, TES Electrical Electronic Corp). Radial scan images at the 12, 3, 6, and 9 o’ clock positions centered over the limbus (local scanning image) and perpendicularly over the pupil center (panoramic scanning image) were obtained. For the local scanning images, only temporal images were analyzed in the current study. Images were taken directly from the machine output as 1024×655 pixel JPEG files. The size of local scanning image was 9.98 mm×6.38 mm (1024×655 pixels), and the size of panoramic scanning image was 15.60 mm×9.97 mm (1024×655 pixels). Conversions between the length and the number of pixel of the images are: for panoramic scanning image, 1 pixel = 15.2 μm; for local scanning image, 1 pixel = 9.7 μm. Conversions between the area and the number of pixels of the images area are: for panoramic scanning image, 1 pixel = 231.9 μm^2^; for local scanning image, 1 pixel = 94.9 μm^2^.

Each image was analyzed by two trained physicians (WZ and LX, who were masked to the clinical data) on two occasions two weeks apart in order to minimize the influence of observer memory on assignment of scleral spur position. Observer 1 (WZ) had been doing general UBM measurements for 3 years and trained for UBM measurements with Photoshop on 100 images. Observer 2 (LX) was trained for UBM measurements with Photoshop on 30 images.

### Image processing and definition of anterior segments area parameters

**Fig 1:**
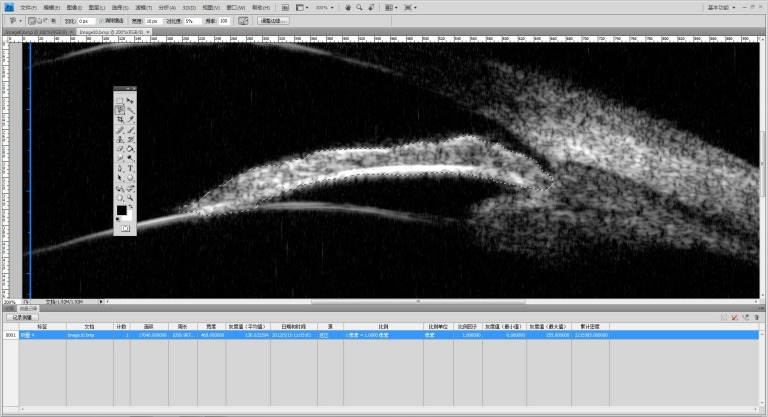
Example of the area calculation by Photoshop. The size of the area in question was calculated by counting the number of pixels enclosed in the area automatically.

Photoshop software (Adobe Photoshop CS4, Adobe Systems Inc, CA, USA) was used for image processing in the current study. To analyze UBM images, observers opened the image in Photoshop and estimated the location of the scleral spurs. Using the “Magnetic Lasso Tool” in Photoshop, the observers drew the boundaries of the area with the boundary detection function in any selected area by Photoshop (S1 Movie). The size of the area in question was calculated by counting the number of pixels enclosed in the area (Fig. 1).

**Fig 2:**
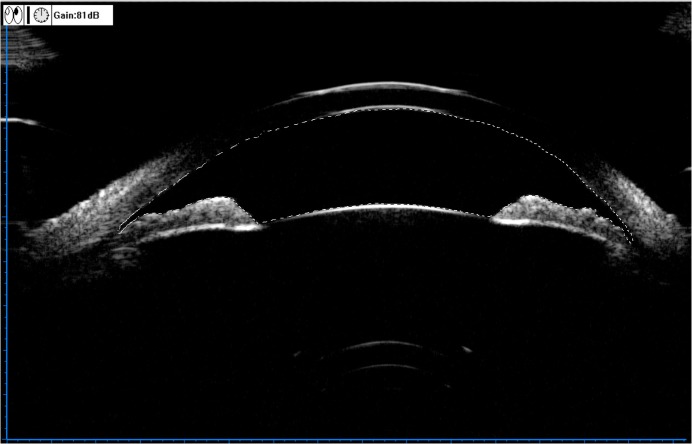
Illustration of the anterior chamber area (ACA), which was defined by the borders of the corneal endothelium, angle, iris surface, and the anterior lens epithelium.

**Fig 3:**
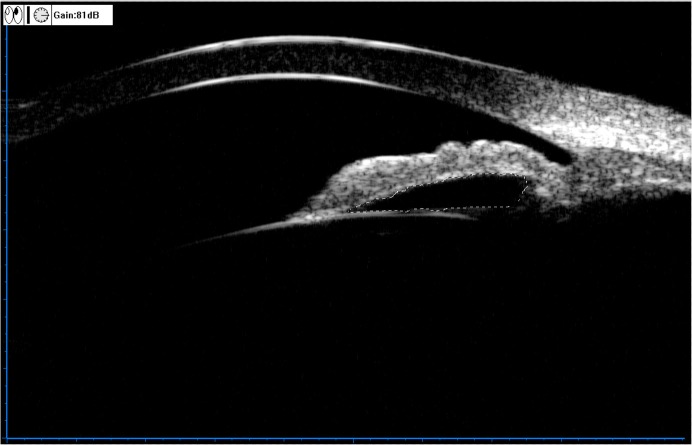
Illustration of the posterior chamber area (PCA), which was defined by the borders of the posterior iris surface, lens zonules, and the posterior border of the lens.

**Fig 4:**
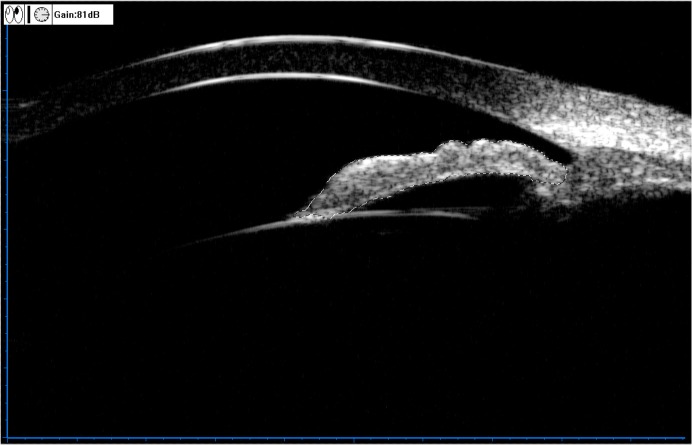
Illustration of the iris cross-section area (ICA), which was bound by the anterior and the posterior surfaces of the iris and the root of the iris.

**Fig 5:**
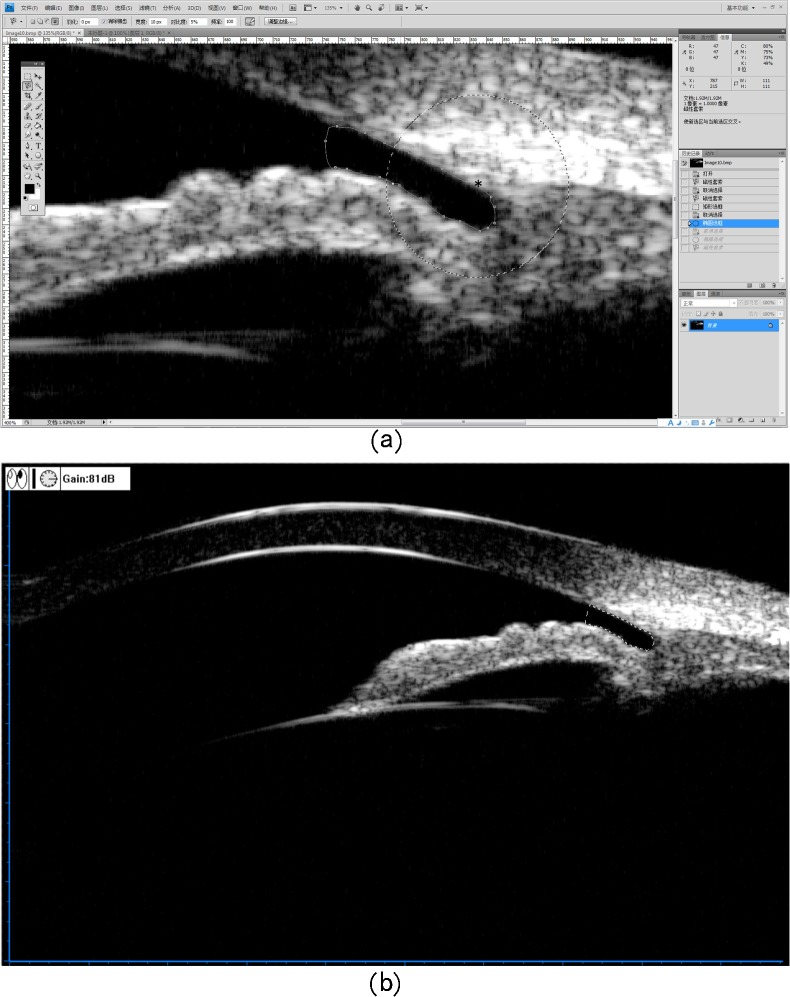
Illustration of the angle recess area (ARA). Define the borders of the corneal endothelium and the anterior iris surface, and define the scleral surface junction point as the spur, then draw a circle centered on the spur with diameter of 750 μm (154 pixels) (a). The enclosed area is the ARA (b).

The “Magnetic Lasso” Tool in Photoshop was used to define the anterior chamber area (ACA) by drawing the borders of the corneal endothelium, angle, iris surface, and the anterior lens epithelium (Fig. 2). The posterior chamber area (PCA) was defined by the borders of the posterior iris surface, lens zonules, and the anterior border of the lens (Fig. 3). The iris cross-section area (ICA) was bound by the anterior and the posterior surfaces of the iris and the root of the iris (Fig. 4). The angle recess area (ARA) defined by Ishikawa et al [10] was as the following: first define the borders of the corneal endothelium and the anterior iris surface, and then define the scleral surface junction point as the spur, then draw a circle centered on the spur with diameter of 750 μm (154 pixels) (Fig. 5-A). The enclosed area was the ARA (Fig. 5-B).

The parameters of ACA, PCA, ICA, and ARA measured by each observer were recorded. The scleral spur location of each image on pixel coordinates defined by two observers on each separate occasion was also recorded.

A single observer performed the measurements on all images at one time. The amount of time the observer spent on the measurements was recorded.

### Statistical methods

Statistical analyses were performed using SPSS software version 13.0 (SPSS, Inc., Chicago, IL). The means and standard deviations of the anterior segment parameters were calculated. For the intraobserver reproducibility, the 1^st^ and 2^nd^ measurements were used. For the interobserver reproducibility, the 1^st^ measurements of each observer were used. Limits of agreement (LoA) were defined as the mean±1.96 standard deviations (SD) of the differences. The coefficient of variation (CV) was defined as the standard deviation of differences divided by the overall mean. Paired *t* tests were used for the differences between repeated measurements. *P*<0.05 was considered statistically significant.

## Results

**Fig 6:**
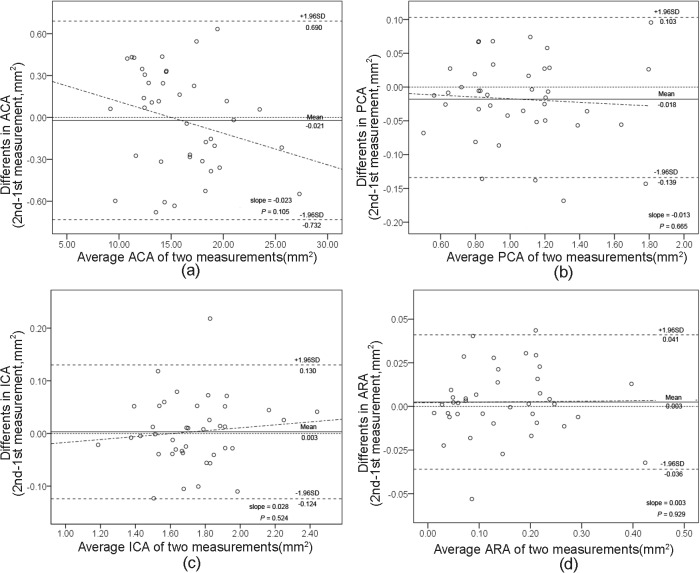
Bland-Altman plots of intraobserver reproducibility of UBM measurements using Photoshop software by observer 1. Measurements of anterior chamber area (ACA) (a), posterior chamber area (PCA) (b), iris cross-section area (ICA) (c), and angle recess area (ARA) (d). The difference is calculated as the 2nd measurement minus the 1st measurement. The grey dashed line represents regression line of difference between 1st and 2nd measurements.

**Fig 7:**
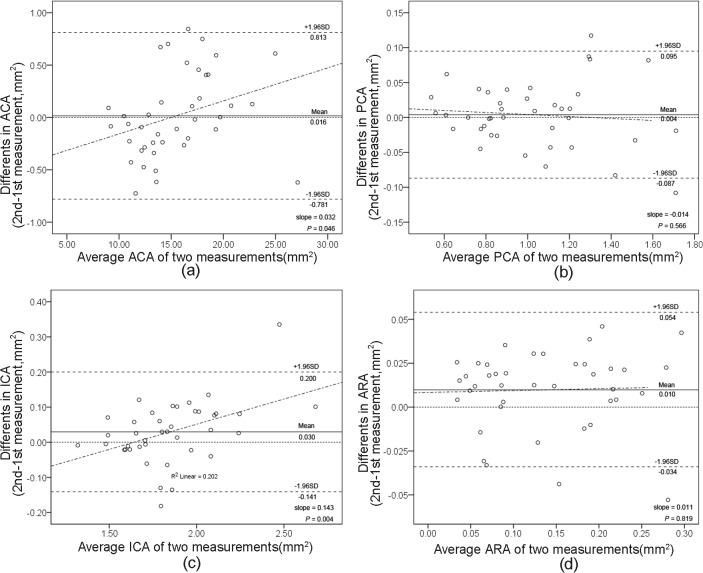
Bland-Altman plots of intraobserver reproducibility of UBM measurements using Photoshop software by observer 2. Measurements of anterior chamber area (ACA) (a), posterior chamber area (PCA) (b), iris cross-section area (ICA) (c), and angle recess area (ARA) (d). The difference is calculated as the 2nd measurement minus the 1st measurement. The grey dashed line represents regression line of difference between 1st and 2nd measurements.

As shown in Table 1, the intraobserver reproducibilities of UBM measurements using Photoshop software were good, with no more than 5% coefficient of variation (CV) and more than 0.95 intraclass correlation coefficient (ICC) for most measurements (Fig. 6-A, Fig. 6-B, Fig. 6-C, and Fig. 7-A, Fig. 7-B, Fig. 7-C). Since ARA was smaller than the other parameters, ICCs of ARA tended to be lower. However, the upper or lower limit values of ARA were not more than 1/5 of the overall mean values in both normal controls and narrow/closed angle subjects (Fig. 6-D and Fig. 7-D).

**Table 1:** Intraobserver reproducibilities of UBM measurements using Photoshop software.

		Observer 1					Observer 2				
		First (mm^2^)	Second(mm^2^)	LoA (mm^2^)	CV (%) (Range)	ICC	First (mm^2^)	Second (mm^2^)	LoA (mm^2^)	CV (%) (Range)	ICC
**All**	**ACA**	15.937±4.217	15.916±4.123	−0.732∼0.690	1.4 (0∼4.0)	0.996	15.477±4.032	15.492±4.161	−0.781∼0.813	1.5 (0∼4)	0.995
	**PCA**	1.061±0.334	1.043±0.330	−0.139∼0.103	3.3 (0∼11.0)	0.982	1.017±0.308	1.021±0.304	−0.087∼0.095	2.3 (0∼7)	0.989
	**ICA**	1.730±0.241	1.733±0.247	−0.124∼0.130	2.0 (0∼8.0)	0.966	1.833±0.256	1.862±0.295	−0.141∼0.200	2.4 (0∼10)	0.946
	**ARA**	0.145±0.099	0.148±0.099	−0.036∼0.041	9.8 (0∼5.1)	0.981	0.133±0.078	0.142±0.078	−0.034∼0.054	14.3 (0∼53)	0.952
**Normal control**	**ACA**	19.232±3.285	19.074±3.330	−0.838∼0.522	1.2 (0∼3)	0.994	18.505±3.334	18.723±3.256	−0.560∼0.996	1.4 (0∼4)	0.991
	**PCA**	0.909±0.245	0.888±0.244	−0.133∼0.090	3.7 (0∼11)	0.971	0.870±0.238	0.882±0.232	−0.064∼0.088	2.5 (0∼7)	0.986
	**ICA**	1.751±0.284	1.772±0.291	−0.112∼0.155	2.0 (0∼8)	0.971	1.872±0.305	1.916±0.337	−0.065∼0.153	2.0 (0∼5)	0.977
	**ARA**	0.221±0.081	0.224±0.076	−0.035 ∼ 0.042	5.5 (0∼15)	0.970	0.195±0.053	0.208±0.051	−0.033∼0.059	8.4 (1∼17)	0.873
**Narrow/Closed angle subjects**	**ACA**	12.642±1.692	12.758±1.740	−0.535 ∼ 0.767	1.7 (0∼4)	0.980	12.449±1.718	12.262±1.721	−0.794∼0.421	1.6 (0∼4)	0.979
	**PCA**	1.212±0.348	1.197±0.337	−0.147 ∼ 0.118	2.9 (0∼9)	0.981	1.164±0.304	1.160±0.308	−0.108∼0.100	2.2 (0∼6)	0.986
	**ICA**	1.708±0.193	1.694±0.194	−0.127 ∼ 0.097	2.0 (0∼6)	0.956	1.794±0.196	1.809±0.241	−0.200∼0.230	2.9 (0∼10)	0.880
	**ARA**	0.070±0.039	0.072±0.454	−0.037 ∼ 0.040	14 (2∼51)	0.894	0.070±0.037	0.077±0.031	−0.035∼0.048	20.1 (0∼53)	0.796

**ACA**: anterior chamber area

**PCA**: posterior chamber area

**ICA**: iris cross-section area

**ARA**: angle recess area

**LoA**: 95% limit of agreement

**CV**: coefficient of variation

**ICC**: intraclass correlation coefficient.

**Fig 8:**
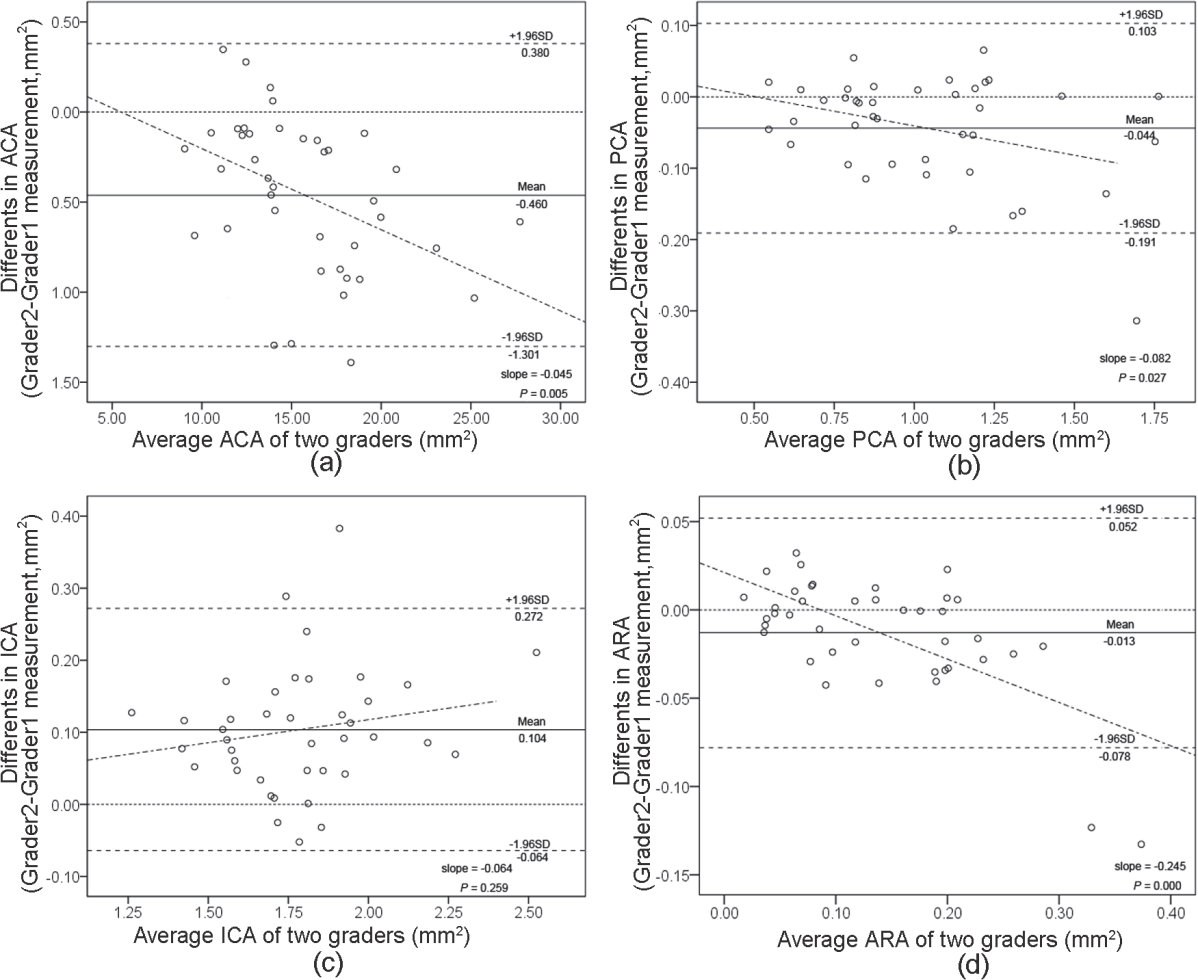
Bland-Altman plots of interobserver reproducibility of UBM measurements using Photoshop software by observer 1 and observer 2. Measurements of anterior chamber area (ACA) (a), posterior chamber area (PCA) (b), iris cross-section area (ICA) (c), and angle recess area (ARA) (d). The difference is observer 2’s measurement minus observer 1’s measurement. The grey dashed line represents regression line of difference between the measurements of observer 1 and observer 2.

The method also showed great interobserver reproducibilities, with no more than 5% CV in the measurements of ACA, PCA, and ICA (Table 2, Fig. 8-A, Fig. 8-B and Fig. 8-C). While the CVs of ARA were slightly larger, they were still within 20% (Table 2, Fig. 8-D). Similarly, the intraobserver and interobserver reproducibilities for defining the spur location had more than 0.97 ICCs (Table 3).

**Table 2:** Interobserver reproducibilities of UBM measurements using Photoshop software.

		Observer 1 (mm^2^)	Observer 2 (mm^2^)	LoA (mm^2^)	CV (Range) (%)	ICC
**All**	**ACA**	15.937±4.217	15.477±4.032	−1.301 ∼ 0.380	2.2(0–7)	0.989
	**PCA**	1.061±0.334	1.017±0.308	−0.191 ∼ 0.103	3.8(0–13)	0.964
	**ICA**	1.730±0.241	1.833±0.256	−0.064 ∼ 0.272	4.3(0–14)	0.867
	**ARA**	0.145±0.099	0.133±0.776	−0.078 ∼ 0.052	12.8(0–41)	0.922
**Normal control**	**ACA**	19.232±3.285	18.505±3.334	−1.486 ∼ 0.032	2.8(0–7)	0.970
	**PCA**	0.909±0.245	0.870±0.238	−0.159 ∼ 0.080	4.2(0–12)	0.957
	**ICA**	1.751±0.284	1.872±0.305	−0.049 ∼ 0.293	4.9(0–14)	0.883
	**ARA**	0.221±0.081	0.195±0.053	−0.103 ∼ 0.051	9.3(0–26)	0.784
**Narrow/Closed angle subjects**	**ACA**	12.642±1.692	12.449±1.717	−0.741 ∼ 0.353	1.6(0–5)	0.981
	**PCA**	1.212±0.348	1.164±0.304	−0.222 ∼ 0.125	3.4(0–13)	0.955
	**ICA**	1.708±0.193	1.794±0.196	−0.076 ∼ 0.247	3.8(0–12)	0.833
	**ARA**	0.070±0.039	0.070±0.037	−0.036 ∼ 0.037	16.3(0–41)	0.885

**ACA**: anterior chamber area

**PCA**: posterior chamber area

**ICA**: iris cross-section area

**ARA**: angle recess area

**LoA**: 95% limit of agreement

**CV**: coefficient of variation

**ICC**: intraclass correlation coefficient.

**Table 3:** Intraobserver and interobserver reproducibilities of spur location detection.

		Observer 1			Observer 2			Interobserver		
		Difference (pixel)*	LoA (pixel)	ICC	Difference (pixel)*	LoA (pixel)	ICC	Difference (pixel)†	LoA (pixel)	ICC
**All**	**x**	−0.4±3.9	−8.0∼7.2	1.000	1.3±5.4	−9.3∼11.9	0.999	−2.9±7.7	−18.0∼12.2	0.999
	**y**	0.1±3.7	−7.3∼7.4	0.992	2.2±3.3	−4.3∼8.7	0.990	−1.2±5.7	−12.5∼10.0	0.980
**Normal control**	**x**	−0.3±3.5	−7.1∼6.6	1.000	1.0±6.4	−11.7∼13.6	0.999	−2.1±8.6	−19.1∼14.9	0.999
	**y**	−0.2±2.0	−4.2∼3.8	0.998	2.5±2.9	−3.2∼8.2	0.991	0.1±5.1	−9.9∼10.1	0.985
**Narrow/Closed angle subjects**	**x**	−0.6±4.3	−9.0∼7.9	1.000	1.7±4.3	−6.7∼10.1	1.000	−3.7±6.8	−17∼9.6	0.999
	**y**	0.4±4.9	−9.3∼10.0	0.986	2.0±3.7	−5.3∼9.3	0.990	−2.6±6.2	−14.6∼9.5	0.975

*: difference between 1^st^ measurement and 2^nd^ measurement

**†**: difference between two observers

**x**: value on horizontal axis

**y**: value on vertical axis

**LoA**: 95% limit of agreement

**ICC**: intraclass correlation coefficient.

Although the operating times for both observers were less than 3 minutes per image, there was significant difference in the measuring time between two observers (*p*<0.001, Table 4). Observer 1 was more experienced and needed less time. Moreover, the intraobserver reproducibilities of observer 1 appeared to be better than those of observer 2, with less CV and greater ICC (Table 1). While there was no statistical difference in measuring time between normal control eyes and eyes with narrow/closed angle (*p*>0.1), the latter group appeared to require longer time (Table 4).

**Table 4:** Mean time consumed for measurement on each image of the two observers.

	Observer 1	Observer 2	*p1*
	(second)	(second)	
**All**	133.1±28.0	148.5±31.7	<0.001
**Normal control**	111.7±19.0	127.0±25.0	0.005
**Narrow/Closed angle subjects**	154.6±16.7	169.9±21.7	0.005
***p2***	0.555	0.425	/

*p1*: comparison between observer 1 and observer 2

*p2*: comparison between normal control and narrow/closed angle subjects.

## Discussion

With advances in technology, detailed and objective imaging of the anterior segment has become possible. However, quantitative measurements of the anterior segment can be further improved. In the current study, we introduced a novel method to perform area measurements using Photoshop software. The method was easy to adopt and time saving. Moreover, the intraobserver and interobserver reproducibilities of the measurements were excellent.

Clinical use of UBM facilitates linear quantitative measurements of the anterior segment structures in vivo, which improved our understanding of angle closure [1]. The intraobserver and interobserver agreements of several linear parameters of anterior segment have been proven to be good [2,11]. Anterior segment optical coherence tomography (AS-OCT) followed a similar trajectory and success in terms of usefulness [12]. As our understanding of angle closure grew, the built-in software in UBM and AS-OCT were not sufficient for the analysis of anterior segment. Various image analysis software for anterior segment measurements have been introduced [7,8,13], yet they could not be widely adopted because of software incompatibility on different instruments. Moreover, most analyses could only be performed on linear measurements. Finally, it was difficult to avoid considerable error in the automated algorithms of these software.

Photoshop is an easily-accessible and widely-used software, and has been applied to the measurements of fundus lesions [14–16]. With powerful function of boundary detection in any selected area, Photoshop can carry out semi-automated area measurements easily in a short time. In the current study, Photoshop could automatically trace and draw the boundaries of a region and calculate the area, under the guidance of the observer. The intraobserver and interobserver agreements of the anterior segment measurements by Photoshop were good, with no more than 5% coefficient of variation (CV) and more than 0.95 intraclass correlation coefficient (ICC) in most measurements.

In the current study, observer 1 is an experienced UBM user while observer 2 is a beginner with training on only thirty images before entering the study. It is not surprising that observer 1 spent less time on the measurements and had better intraobserver reproducibilities. However, the interobserver reproducibilities were satisfactory, and the operating times for both observers were short (less than 3 minutes per image).

Some anterior segment measurements such as ARA require manual identification of the scleral spur as a measurement reference point. Subjective identification of the scleral spur would likely increase the variance of measurements [2,17]. Previous studies described newly developed algorithms and measurement methods for images from AS-OCT and UBM, both of which could perform automated measurement on the reference of a subjective identification of the scleral spur [7,13]. However, good-quality UBM images with high signal-to-noise ratio are required to identify the edge points correctly [7]. The limited resolution and poor contrast of the angle recess area in AS-OCT images may compromise accuracy. The measurements taken in eyes with very narrow angles or iridotrabecular contact can be problematic if the software identifies the apparent contact between the peripheral iris and trabecular meshwork as the reference point [13]. Given these problems in automated measurements, semiautomatic measurement remains indispensable. In the current study, the intraobserver and interobserver reproducibilities were great for the spur location detection, compared with a previous study on the AS-OCT [13]. This may be because the locations of the scleral spur on UBM images are clear and easily identifiable, especially by trained observers. In addition, we found that reproducibility varied little between wide and narrow/closed angles in the current study, which was different from a previous study using automated software on images from AS-OCT [13].

In summary, UBM images can be analyzed reproducibly with Photoshop software. This method, with a relatively steep learning curve, provides valuable information beyond linear measurements that may further elucidate pathogenesis of anterior segment diseases. More studies with Photoshop on other image formats may further validate its usefulness in image processing.

## Supporting Information

S1 Movie Illustration of analyzing UBM image using Photoshop. (MOV)
